# Periodontitis biomarkers through thermal desorption-gas chromatography-mass spectrometry analysis

**DOI:** 10.3389/fdmed.2025.1684773

**Published:** 2025-11-24

**Authors:** Qin Liang, Yanling Zhang, Xiaoli Zhang, Yizhou Liu, Shaojia Xu, Yajuan Lei, Xiaodong Li, Chao Yuan

**Affiliations:** 1 Department of Preventive Dentistry, Peking University School and Hospital of Stomatology & National Center for Stomatology & National Clinical Research Center for Oral Diseases & National Engineering Research Center of Oral Biomaterials and Digital Medical Devices & Beijing Key Laboratory of Digital Stomatology & NHC Key Laboratory of Digital Stomatology & NMPA Key Laboratory for Dental Materials, Beijing, China; 2 Department of Periodontology, Peking University School and Hospital of Stomatology & National Center for Stomatology & National Clinical Research Center for Oral Diseases & National Engineering Research Center of Oral Biomaterials and Digital Medical Devices& Beijing Key Laboratory of Digital Stomatology & NHC Key Laboratory of Digital Stomatology & NMPA Key Laboratory for Dental Materials, Beijing, China; 3 Shimadzu (China) Co., LTD. China Innovation Center, Beijing, China

**Keywords:** salivary volatile metabolites, periodontitis, biomarker, TD-GC-MS, oralexhaled breath

## Abstract

**Background:**

Periodontitis is a growing public health concern worldwide. Salivary volatile metabolites have emerged as promising biomarkers for the diagnosis of periodontal disease. However, research on the collection and identification of these metabolites in periodontitis patients remains limited.

**Objectives:**

To explore methods for collecting and identifying salivary volatile metabolites in periodontitis patients and investigate their potential as biomarkers for diagnosing periodontal disease.

**Method:**

Oral exhaled breath and saliva samples were collected from 115 periodontitis patients and 35 healthy individuals, divided into four cohorts. The discovery cohort (Periodontitis: *P* = 55, Healthy: *H* = 23) and the test cohort (*P* = 48, *H* = 23) were screened and validated for potential biomarkers in volatile metabolites from oral exhaled breath by thermal desorption-gas chromatography-mass spectrometry (TD-GC-MS). The validation cohort 1 (*P* = 12, *H* = 12) was tested for volatile metabolites in saliva by solid-phase microextraction-gas chromatography-mass spectrometry (SPME-GC-MS), while validation cohort 2 (*P* = 55, *H* = 23) was tested for metabolic pathways in saliva by liquid chromatography-mass spectrometry (LC-MS).

**Result:**

A total of 78 Volatile organic compounds (VOCs) were detected by TD-GC-MS, with 14 differential VOCs identified. A diagnostic model was established using cyclohexanone, styrene, and ethanol, yielding a combined AUC of 0.8237. These metabolites were also detected in saliva by SPME-GC-MS, with cyclohexanone showing higher expression in the periodontitis group (*P* < 0.05). The caprolactam degradation pathway was a key source of volatile metabolites in the oral exhaled breath of periodontitis patients.

**Conclusion:**

We developed a novel method for analyzing salivary volatile metabolites using TD-GC-MS, demonstrating potential for periodontitis diagnosis. Cyclohexanone is identified as a potential biomarker for periodontitis, and the caprolactam degradation pathway may play a significant role in future studies on oral microbiota dysbiosis in periodontitis patients.

## 1 Introduction

Periodontitis is a chronic infectious disease induced by dental plaque microorganisms, characterized by inflammation and destruction of periodontal supporting tissues, which may ultimately lead to tooth loss (1, 2). According to the Global Burden of Disease study, in 2017, severe periodontitis affected approximately 71.48 million people worldwide, with a disability-adjusted life year rate of 63.49 per 100,000 person-years. This represents a 6.01% increase since 1990, with a notably higher disease burden observed in Asia. Therefore, it is crucial to prioritize the prevention and management of periodontal disease (3).

In recent years, the utilization of biomarkers for early non-invasive diagnosis has become a prominent research focus (4–10). Volatile metabolites in saliva and oral exhaled breath, owing to their accessibility and completely non-invasive nature, demonstrate significant diagnostic potential (5, 11). Recent advances have demonstrated the significant potential of volatile metabolites as biomarkers for various systemic conditions, including respiratory diseases (such as lung cancer, asthma, and COPD) (12–14), digestive disorders (e.g., gastric diseases and Crohn's disease (15, 16), and endocrine disorders like obesity and diabetes (17), showing promise for improving disease diagnosis and developing novel biomarkers. The oral microbiota, particularly periodontopathogenic bacteria such as Fusobacterium nucleatum and Porphyromonas gingivalis, produce characteristic volatile metabolites during their metabolic processes. *in vitro* studies have confirmed that these periodontal pathogens generate specific VOCs, including volatile sulfur compounds (e.g., methyl mercaptan and hydrogen sulfide), short-chain fatty acids, indole, and pyridine. These microbe-derived VOCs can diffuse from periodontal pockets into saliva and are subsequently released into oral exhaled breath, thereby providing a direct basis for non-invasive diagnostic approaches (18–20). One study also found elevated levels of volatile sulfur compounds (VSCs) and pyridine in the salivary volatile metabolites of periodontitis patients compared to healthy individuals (21). Saliva contains microorganisms and their metabolites, which interact with the body, and some of these metabolites are released as gases into the oral exhaled breath. A team of researchers has shown that Oral volatile metabolites involve a prominent oral source and that the potential impact of volatiles originating from the oral cavity should be considered in respiratory biomarker studies (22). However, there has been limited research on characterizing salivary volatile metabolites in oral exhaled breath of patients with periodontitis, highlighting the need for further investigation in this area.

The identification and analysis of volatile compounds primarily rely on gas chromatography (GC), mass spectrometry (MS), and their combined applications. Thermal Desorption (TD) is a widely used technique that utilizes a sorbent-containing device to capture and concentrate volatile metabolites before their introduction into the gas chromatograph. This method is particularly prevalent for collecting exhaled breath samples. During analysis, the sorbent is heated to enhance the volatility of the trapped compounds, facilitating their efficient separation and detection within the gas chromatograph (23). In this study, we analyzed oral volatile metabolites from periodontitis patients and healthy individuals using TD-GC-MS. Our objective was to explore methods for the collection and identification of salivary volatile metabolites in patients with periodontitis, and to investigate the potential of these metabolites as biomarkers for the diagnosis of periodontal diseases. Therefore, we modified the previous sampling method of exhaled breath, and further applied TD-GC-MS to the detection of salivary volatile metabolites, using the traditional solid-phase microextraction-gas chromatography-mass spectrometry (SPME-GC-MS) to validate the volatile metabolites of saliva, combined with the validation of relevant metabolic pathways by LC-MS.

## 2 Methods

### 2.1 Study population

The study was registered in Chinese Clinical Trial Registry (Registration Number: ChiCTR2300069047) on March 6, 2023 and was approved by the Local Ethics Committee of Peking University School of Stomatology and Stomatology Hospital (PKUSSIRB-202281149). Population inclusion criteria: a. Age 20–70 years old; b. Having at least 20 teeth in the mouth (excluding the third permanent molar) c. No systemic diseases; e. Signing informed consent; Population exclusion criteria: a. Presence of serious systemic diseases (kidney disease, rheumatoid arthritis, liver dysfunction, stroke or history of stroke) b. Being in the period of pregnancy or breastfeeding c. Presence of periapical abscesses, periodontal abscesses and other active oral infections; presence of oral mucosal disease; severe untreated caries in the mouth: wearing orthodontic appliances or removable dentures; d. History of antibiotic or immunization-related medication use in the past 3 months, e. History of periodontal therapy within 6 months.

### 2.2 Clinical examination and periodontal diagnosis

After obtaining a medical and dental history and a consent form, clinical examinations of all participants were performed by one specialized dentist using manual periodontal probes (PCPUNC 15; HuFriedy Mfg. Co., Inc., Chicago, IL, USA). The clinical periodontal indices, including probing depth (PD), and bleeding index (BI), were measured at six sites per tooth (mesio-buccal, mid-buccal, disto-buccal, mesio-lingual, mid-lingual, and disto-lingual) (24). Subjects were included in different groups based on periodontal examination. Periodontitis patients was required to fulfill as diagnosis of stage II-III extensive periodontitis with reference to the 2018 International Consensus on New Classification of Periodontal and Periimplant Diseases. Healthy individuals was required to have all periodontal pockets probed at a depth of <4 mm and percentage of bleeding on probing ≤10% (25).

### 2.3 Experimental method

**Figure 1:**
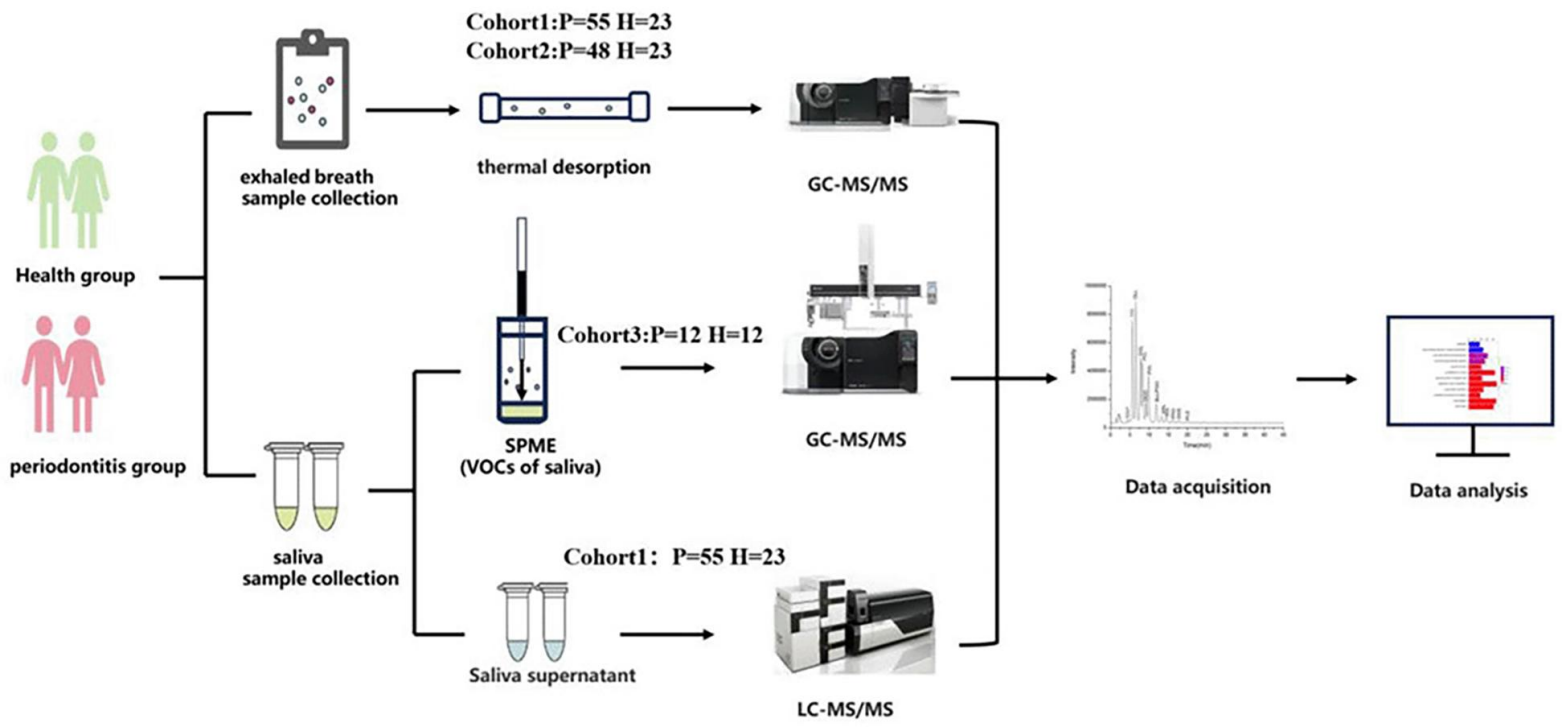
Flow diagram of the study design.

We divided the included periodontitis patients and periodontally healthy individuals into four cohorts: discovery cohort, test cohort, validation cohort 1, and validation cohort 2. The discovery cohort and test cohort screened and validated the potential biomarkers in oral exhale breath by using TD-GC-MS. The validation cohort 1 further detected volatile metabolites in saliva by SPME-GC-MS. The validation cohort 2 tested for the metabolic pathway in saliva by LC-MS. Therefore, in our study, we developed a set of detection and validation methods that can be applied to the oral volatile metabolites of saliva (Figure 1).

### 2.4 Sample collection and preparation

Sampling was conducted between 9:00 and 11:00 a.m. Following an overnight fast, participants were required to abstain from all oral hygiene procedures, food and water intake, gum chewing, and smoking for at least 2 h prior to sample collection. Immediately before sampling, subjects rinsed their mouths with water and subsequently rested for 10 min.

Participants were asked to collect 2–3 mL of non-irritating saliva. The whole saliva was stored without any processing for SPME-GC-MS analysis. The collected saliva was centrifuged (10,000 g, 4°C, 10 min), and the supernatant was retained for subsequent LC-MS analysis.

In addition, we have established a method for collecting the salivary volatile metabolites. Participants were instructed to swell checks to fill the mouth with gas as much as possible, wait for 10–15 s and then exhale all the gas into the Tedlar collection bag. The motion was repeated until the collection bag was full. Nasal inhalation was avoided prior to the exhaled gas collection.

Before collecting gas sample, the sampling bags were repeatedly cleaned by filling with nitrogen and heating to 60℃ for 6 h to remove the residual contaminants. Each sample was transferred to the Tenax-TA trap tubes (Shimadzu, Japan) by pumping 800 mL from the sampling bag to the tube at 150 mL/min. The pumping process was completed by the mini-pump MP-W5P (Shimadzu, Japan). During this process, the VOCs in exhaled breath were trapped in the sorbent tubes. The tubes were sealed and stored at 4 ℃ until analyzed.

### 2.5 Laboratory analysis

#### 2.5.1 Gas sample analysis by TD-GC-MS

To ensure data quality and control for analytical variability, all TD-GC-MS analyses incorporated Toluene-D8 as an internal standard, which was added to sample tubes prior to thermal desorption for instrument response correction and data normalization, thus minimizing batch-to-batch variations.

The gas sample analysis was performed on GCMS-TQ8050 NX (Shimadzu, Japan) coupled with TD-30R thermal desorption (TD) instrument (Shimadzu, Japan). The trapped VOCs in the gas sample were desorbed at 260 ℃ under a flow of 60 mL/min for 5 min and adsorbed on a cold trap with a temperature of −20 °C. 2103;. Then the compounds were re-desorbed at 260 ℃ for 2 min and transfer to the GC inlet. The temperature of inlet and interface was 250 °C. 2103;. Chromatographic separation was performed on the SH-Rxi-624 fused silica capillary column (30 m × 0.25 mm × 1.4 μm, Shimadzu, Japan). Helium was used as the carrier gas with a constant linear velocity of 50 cm/s. The split ratio was 1:10. The GC temperature program was set as follows: the initial temperature was held at 35 ℃ for 5 min and ramped to 150 ℃ at a rate of 10 ℃/min and to 260 ℃ at a rate of 30 ℃/min with a hold time of 2 min. An electron ionization source was used with an ionization voltage of 70 eV and the ion source temperature was 250 °C. 2103;. The acquisition mode of data was Q3 scan mode with the m/z range 28–350. Chromatographic integration was performed by utilizing a single quantification ion extracted from the Extracted Ion Chromatogram (EIC). Raw GC-MS data were processed using GCMS solution software (Shimadzu, Japan) for peak integration and quantitative analysis for volatile metabolites in the sample. volatile metabolites were identified by spectral match according to the mass spectrometry library NIST 20-1, NIST 20-2 and NIST 20 s. The peak area was used to evaluate the relative concentrations of VOCs.

#### 2.5.2 Saliva sample analysis by SPME-GC-MS and LC-MS

500 μL saliva sample was added to 20 mL headspace glass vial. The SPME-GC-MS analysis was performed on GCMS-TQ8050 equipped with AOC-6000Plus autosampler (Shimadzu, Japan). The SPME process were conducted by using 1.10 mm divinylbenzene/carboxen/polydimethylsiloxane (DVB/CAR/PDMS) SPME arrow. The saliva samples were incubated at 37 ℃ for 20 min and stirred continuously at 250 rpm. The SPME arrow was then exposed to the headspace for 45 min at 37 ℃ and desorbed at 250 ℃ in splitless mode for 5 min into the GC. The GC and MS conditions were set to the same as gas samples analysis.

100 μL salivary supernatant sample was added to a 1.5 ml centrifuge tube with 400 μL solution [acetonitrile:methanol=1:1(v:v)]containing 0.02 mg/mL internal standard (L-2-chlorophenylalanine) to extract metabolites. The samples were mixed by vortex for 30 s and low-temperature sonicated for 30 min (5 °C, 40 KHz). The samples were placed at −20 °C for 30 min to precipitate the proteins. Then the samples were centrifuged for 15 min (4 °C, 13,000 g). The supernatant was removed and blown dry under nitrogen. The sample was then re-solubilized with 100 µL solution (acetonitrile: water = 1:1) and extracted by low-temperature ultrasonication for 5 min (5 °C, 40 KHz), followed by centrifugation at 13,000 g and 4 °C for 10 min. The supernatant was transferred to sample vials for LC-MS/MS analysis. Details of the LC-MS laboratory tests for saliva samples are given in Appendix 1.

### 2.6 Data analysis

Subject-related information as well as periodontal clinical parameters were analyzed by SPSS 24.0 software (SPSS; Chicago, IL, USA). Quantitative variables were described as mean ± standard deviation (SD), and frequencies or ratios were used for qualitative variables. The Student's t test was used to compare differences between groups, with a *p* value <0.05 defined as statistically significant. In addition, for gender results, the chi-square test was used to detect statistically significant differences. Differences between groups were analyzed by ANOVA, followed by Tukey's test. After performing a combined normalization of the discovery cohort and test cohort data, further data analysis was conducted. The SPME-GC-MS data were normalized using an internal standard method, followed by log10 transformation for analysis. The preprocessed matrix files were analyzed for differences.LC-MS and TD-GC-MS data results were analyzed by principal component analysis (PCA), ortho-least partial squares-discriminant analysis (OPLS-DA). The data matrix obtained by searching database was uploaded to the Majorbio cloud platform (https://cloud.majorbio.com) for data analysis. The performance of the model was screened and evaluated using Receiver Operating Characteristic curves (ROC). SPME-GC-MS results run on the MetaboAnalyst6.0 (https://www.metaboanalyst.ca/) platform.KEGG (https://www.kegg.jp/kegg/tool/map_pathway.html) pathway analysis was performed to identify the metabolic pathways associated with the differential and total metabolites.

## 3 Result

### 3.1 Demographic and clinical data

A total of 138 subjects were recruited from 2023 to 2024 at Peking University Stomatology Hospital, including a total of 115 periodontitis patients and 23 healthy individuals. They were divided into four cohorts as shown in Table 1. There was no significant difference between the periodontitis group and healthy group in terms of the number of remaining teeth, gender (*p* = 0.191 and 0.853, respectively). It is statistically significant difference in age (*p* < 0.001). Regarding their periodontal clinical status, there was a statistically significant difference in periodontal probing depth (PPD), and gingival bleeding index (BI) (*p* value < 0.001).

**Table 1:** The characteristics of the study population.

Cohort	Periodontitis group		Healthy group		*p*-value
	PPD	*N*	PPD	*N*	
Discovery cohort	3.32 ± 0.12^a^	*n* = 55	1.88 ± 0.40^b^	*n* = 23	<0.001
Test cohort	3.39 ± 0.93^a^	*n* = 48	1.88 ± 0.40^b^	*n* = 23	<0.001
Validation cohort 1	3.33 ± 0.61^a^	*n* = 12	1.84 ± 0.18^b^	*n* = 12	<0.001
Validation cohort 2	3.32 ± 0.12^a^	*n* = 55	1.88 ± 0.40^b^	*n* = 23	<0.001

Data were described as mean ± SD. PPD, probing pocket depth; *N*: cohort number. Statistically significant difference (student's t test, *p*-value < 0.001);

a,b Indicate statistically significant differences among groups (*p* < 0.05) as determined by one-way ANOVA followed by Tukey's HSD test.

### 3.2 Characterization of salivary volatile metabolites by TD-GC-MS

#### 3.2.1 Analysis of gas samples

**Figure 2:**
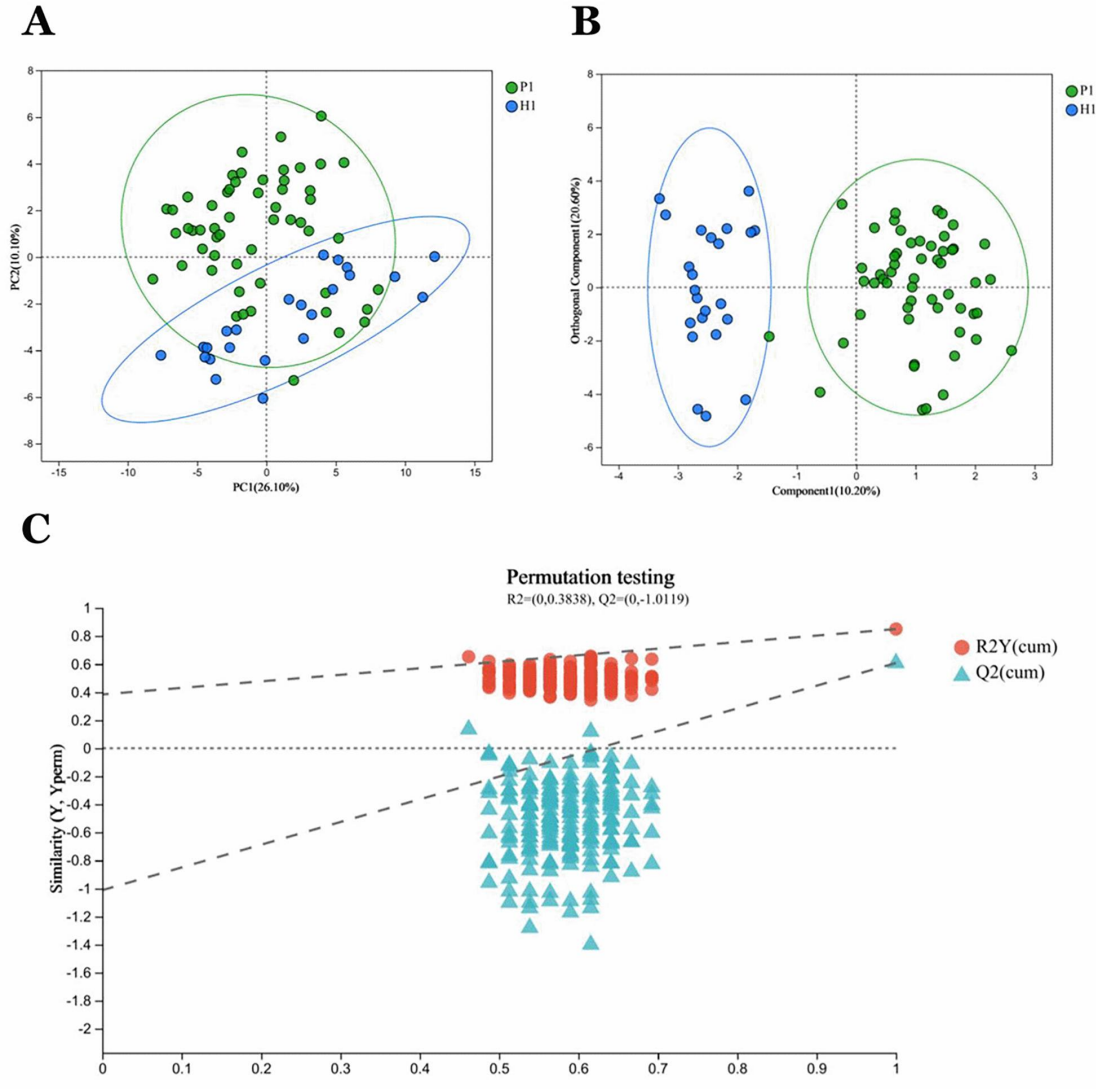
P1 and HI group samples in general. **(A)** PCA analysis of sample relationships. Green dots indicate group P1 samples. Blue dots indicate group H1 samples. **(B)** OPLS-DA analysis of sample relationship. **(C)** Permutation testing of OPLS-DA: R2 = (0,0.3838), Q2 = (0, −1.0119).

Principal Component Analysis (PCA) revealed distinct clustering patterns between sample groups P1 and H1 (Figure 2A). Intra-group samples demonstrated tight clustering, indicating high experimental reproducibility within each group. Conversely, substantial intergroup separation suggested marked metabolic heterogeneity between the two groups. These metabolic differences were further confirmed by Orthogonal Partial Least Squares Discriminant Analysis (OPLS-DA), which showed clear group discrimination (Figure 2B). The OPLS-DA model exhibited robust performance with high explanatory (R^2^Y = 0.85) and predictive (Q^2^ = 0.608) capabilities. To validate model integrity, a 200-iteration permutation test was performed, revealing a negative intercept (*Q*^2^ regression line < 0) that effectively excluded overfitting concerns (Figure 2C).

#### 3.2.2 Differential metabolite screening and pathway analysis

**Figure 3:**
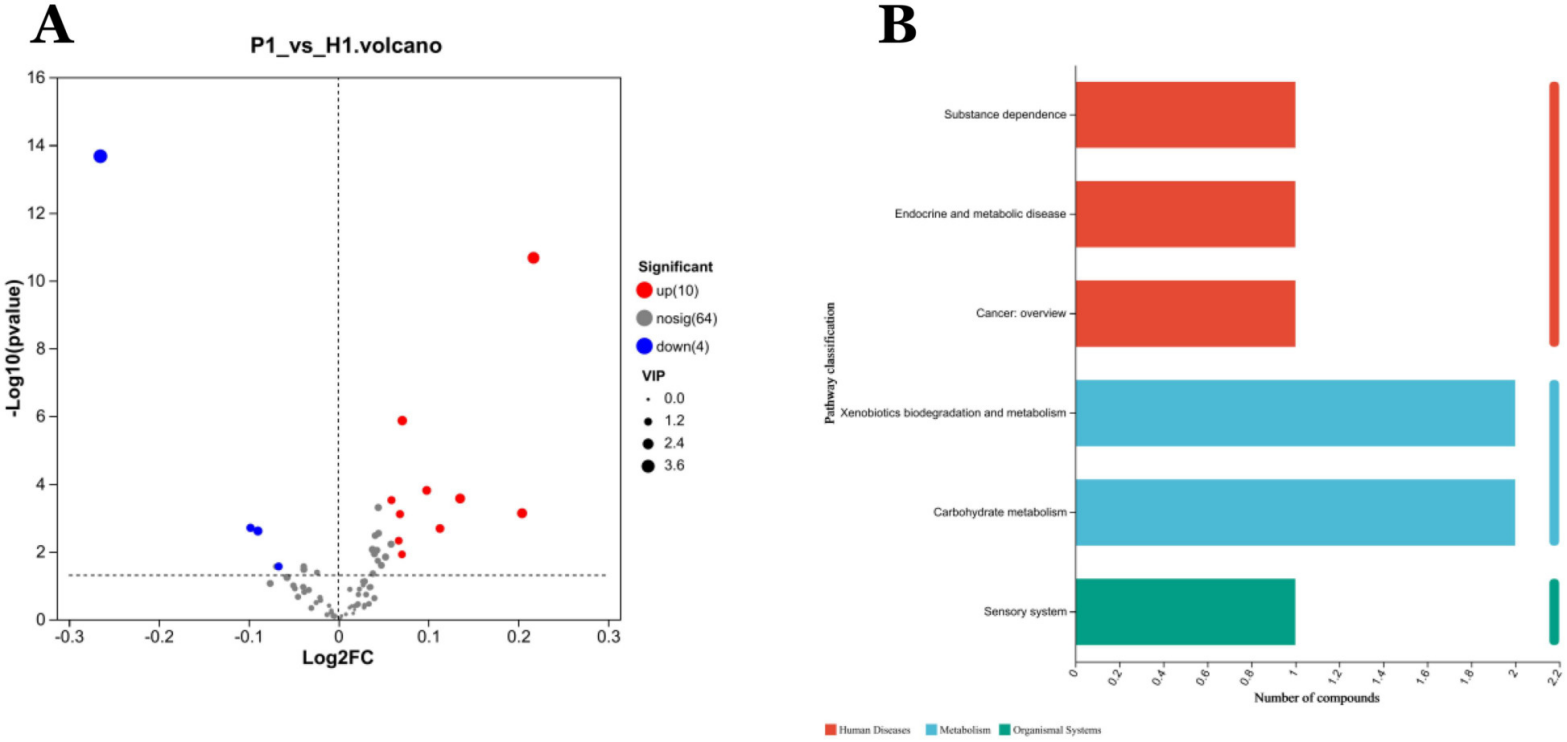
Analysis of differentially expressed metabolites in VOCs. **(A)** Volcano diagram: red spots indicate the upregulated differentially expressed metabolites while blue spots indicate the downregulated differentially expressed metabolites. Gray spots represent metabolites with no significant differential expression. Screening conditions for VIP ≥ 1 and *P*-value ≤ 0.05 for OPLS-DA model. **(B)** Kegg pathway classification: Metabolites are detected and annotated.

**Figure 4:**
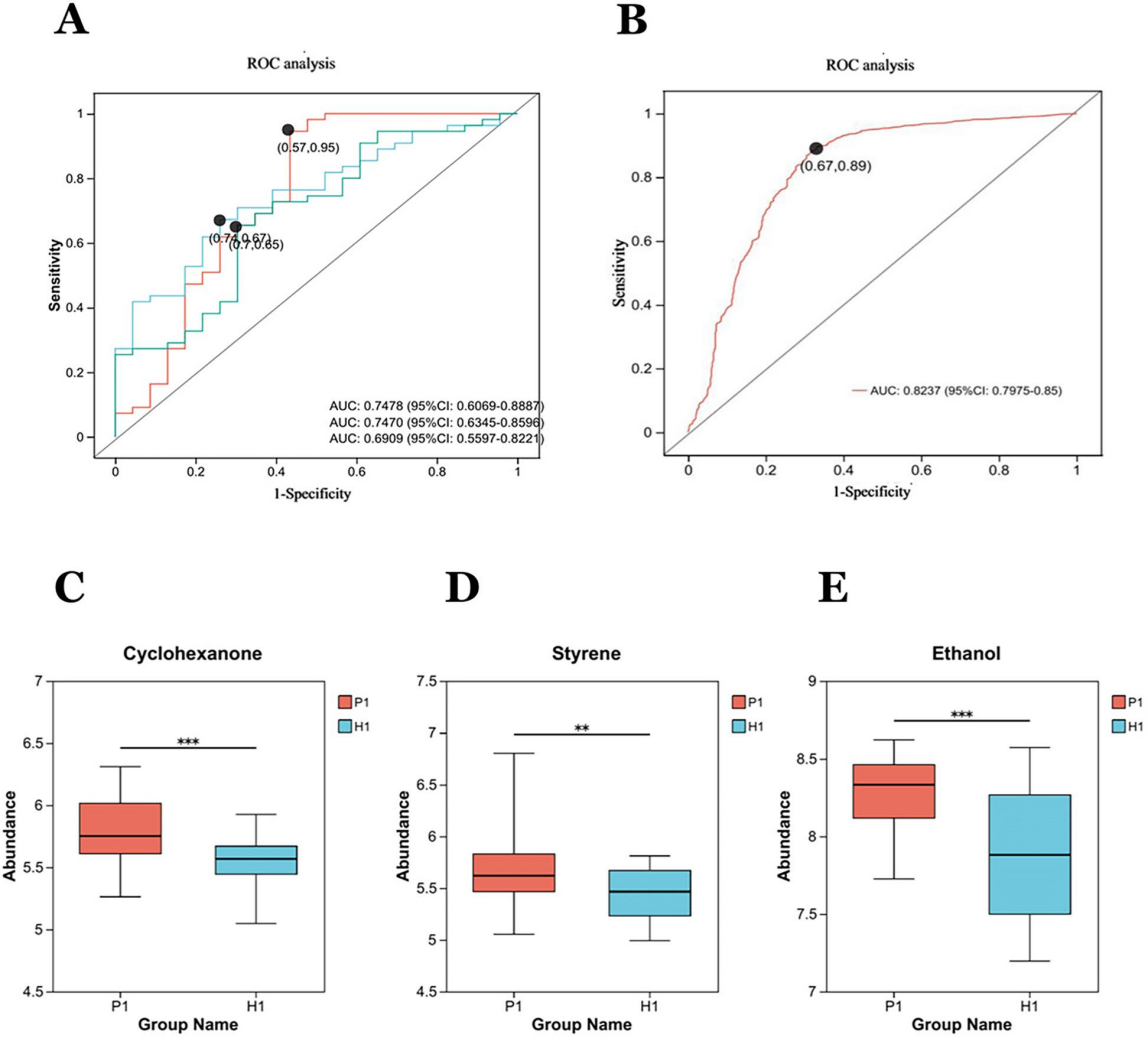
Diagnostic performance of exhaled gas metabolites cyclohexanone, styrene, ethanol, and combined combinations of in a discovery cohort: **(A)** independent receiver operating characteristic (ROC) analysis of cyclohexanone, styrene, ethanol. The areas under the curves (AUC) respectively are 0.7470, 0.6909, 0.7478. **(B)** The AUC for the combination of the three metabolites is 0.8237. **(C–E)** The box diagrams show the comparison of cyclohexanone, styrene, and ethanol the expression levels of between two groups. **Statistically significant difference (student's t test, *p*-value < 0.01). ***Statistically significant difference (student's t test, *p*-value < 0.0001).

A total of 78 VOCs were identified in the discovery cohort. Differential VOCs were selected based on Variable Importance in Projection (VIP) scores from the OPLS-DA model and *p*-values from Student's t-test. VOCs with VIP > 1 and *P* < 0.05 were considered statistically significant (Figure 3A). A total of 14 differential VOCs (2-Ethyl-oxetane, Butanoic acid, methyl ester, 2-Pyrrolidinone, 1-methyl-, Ethanol, 2-butoxy-, Ethanol, Cyclopentane, methyl, Decane, 2,4-Dimethyl-1-heptene, Butylated Hydroxytoluene, Undecane, 3-methyl-, Cyclohexanone, 2,3-Butanedione, Styrene, Propanoic acid, 2-methyl-, 1-(1,1-dimethylethyl)-2-methyl-1,3-propanediyl ester)were identified in our screening. To control for the potential confounding effect of age between the periodontitis group and the healthy control group, univariate linear regression analysis was conducted to assess the relationship between the 14 identified differential metabolites and age in both groups (detailed results are provided in Appendix 1). According to the pre-defined screening criteria (R^2^ > 0.5 and *P* < 0.05), 2-Ethyl-oxetane (R^2^ = 0.537, *P* < 0.05) and Cyclopentane, methyl (R^2^ = 0.512, *P* < 0.05) in the healthy group were excluded due to significant age correlation. Ultimately, 12 metabolites showing no significant association with age were retained as candidate differential markers, including: Butanoic acid, methyl ester, 2-Pyrrolidinone, 1-methyl-, Ethanol, 2-butoxy-, Ethanol, Decane, 2,4-Dimethyl-1-heptene, Butylated Hydroxytoluene, Undecane, 3-methyl-, Cyclohexanone, 2,3-Butanedione, Styrene, Propanoic acid, 2-methyl-, 1-(1,1-dimethylethyl)-2-methyl-1,3-propanediyl ester.As a result, 14 differential VOCs were identified and subsequently mapped to KEGG pathways for further functional annotation. Among them, cyclohexanone, styrene, and ethanol were associated with the “Microbial metabolism in diverse environments” pathway (Figure 3B). Additionally, these three metabolites exhibited significantly higher expression levels in the periodontitis group compared to healthy controls (Figures 4C–E).

#### 3.2.3 Diagnostic capability analysis of candidate differential metabolites

The three metabolites, cyclohexanone, styrene, and ethanol were evaluated by ROC (Figure 4A), as well as a joint ROC analysis was done to discriminate this group of metabolites (Figure 4B). The AUC of cyclohexanone was 0.7470 (95% CI: 0.6345–0.8596), the AUC of styrene was 0.6909 (95% CI: 0.5597, 0.8221), and the AUC of ethanol was 0.7478 (95% CI. 0.6069, 0.8887). The combined AUC of the three metabolites was 0.8237 (95%CI: 0.7975–0.85).The results indicated that the diagnostic model with the three metabolites together had well diagnostic effect.

#### 3.2.4 Secondary validation of the diagnostic model

**Figure 5:**
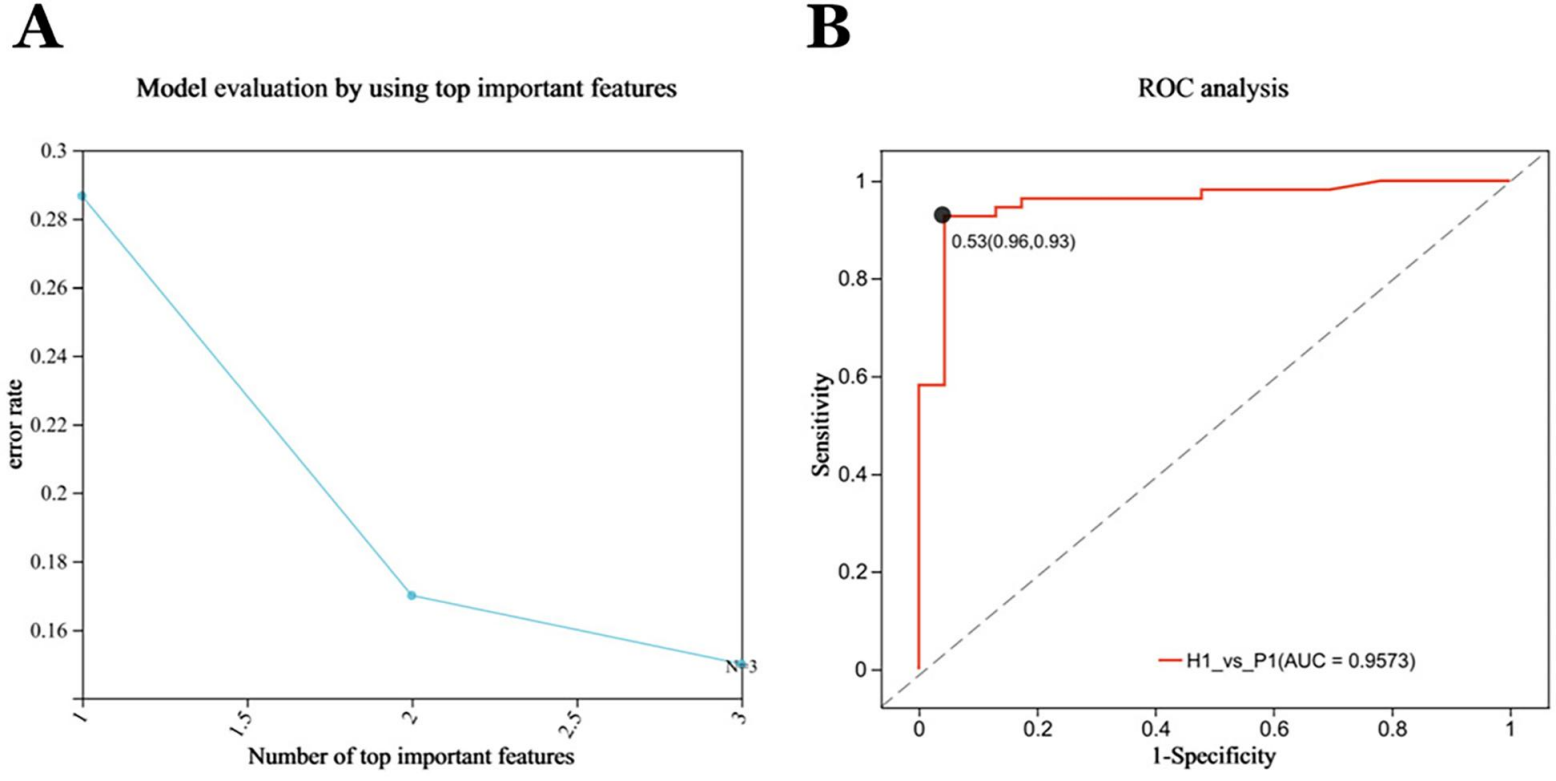
Forty-eight subjects were included in the test cohort for optimization, forming the validation cohort P2 (*n* = 48), H2 (*n* = 23). **(A)** Evaluation of Random Forest Model: The RFECV (Recursive Feature Elimination with Cross-Validation) algorithm was used to compute the features, with the horizontal coordinates representing the number of metabolites (variables) ranked TOP in terms of importance, and the vertical coordinates representing the average prediction error rate when the corresponding number of metabolites (variables) was used; solid points with markers in the plot indicate the point with the lowest error rate selected. The lowest model error rate was found when all three metabolites were modeled together. **(B)** The Independent Receiver operating characteristic (ROC) analysis of the RF models. The random forest models all achieved high accuracy with an AUC of 0.9573.

In the test cohort. The Random Forest (RF) model was used to identify the diagnostic ability of the diagnostic model composed of cyclohexanone, styrene, and ethanol. The results illustrated that when the RF model was constructed with the three metabolites, the model error rate was the lowest (Figure 5A), the AUC of the ROC curve was 0.9573 (Figure 5B), and the Random Forest model achieved a high level of accuracy, again demonstrating the superior diagnostic ability of the three metabolites.

### 3.3 Characterization of salivary volatile metabolites by SPME-GC-MS

**Figure 6:**
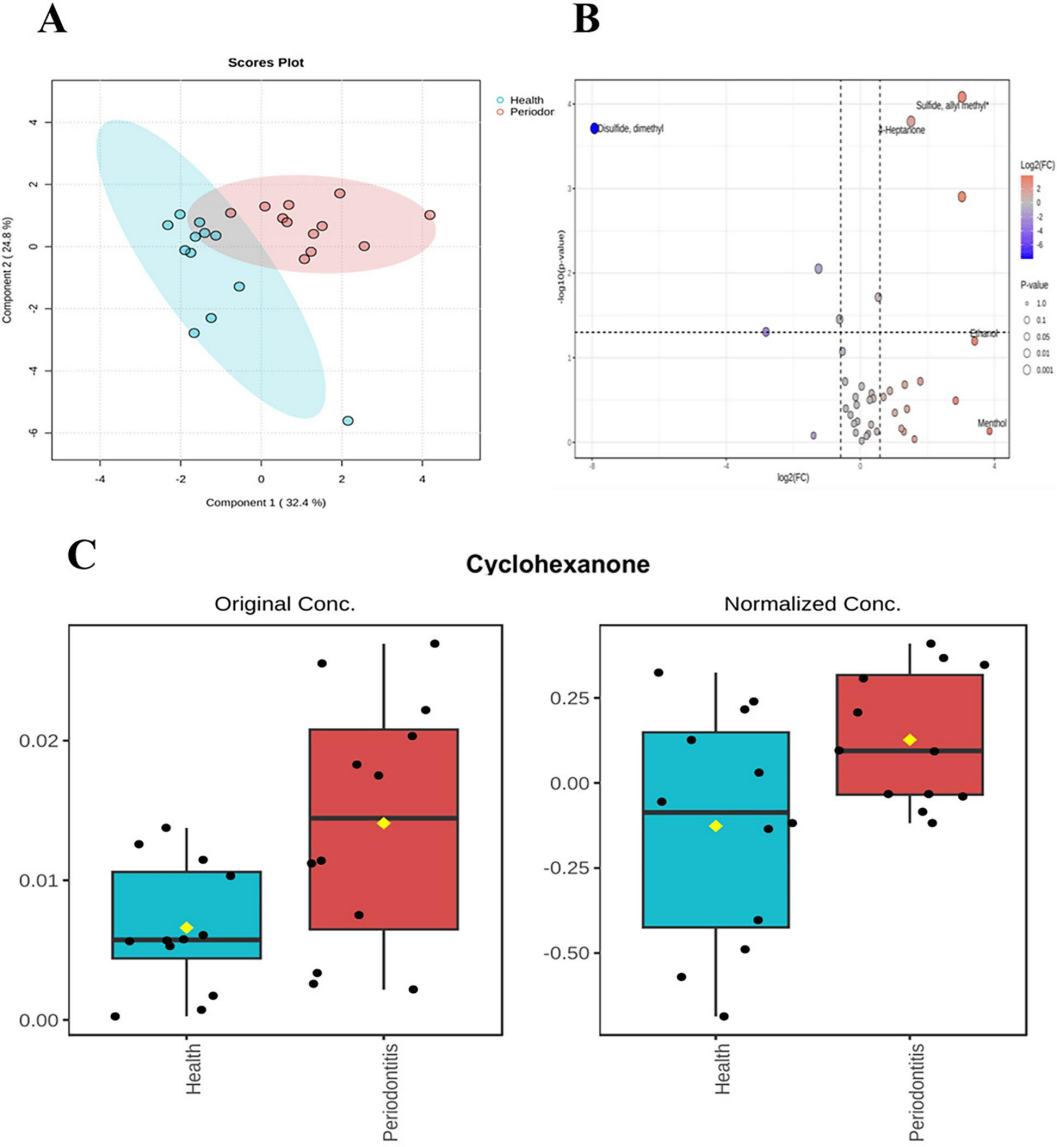
Differences in salivary volatile metabolites. saliva validation cohort 2:The sample consisted of a periodontitis group (*n* = 12) and a healthy group (*n* = 12). **(A)** Unsupervised principal component analysis (PCA) showed that the samples were reproducible within groups, but there were some differences between groups. **(B)** Volcano plot combines results from Fold Change (FC) Analysis and T-tests into one single graph, Mean quantitative differential fold change was limited to ± 1.5, *P* value less than 0.05. **(C)** Histogram of cyclohexanone before and after standardized treatment.

A total of 39 salivary volatile metabolites were identified in Validation Cohort 1 by SPME-GC-MS. Principal Component Analysis (PCA) of the periodontitis group (*n* = 12) and healthy group (*n* = 12) revealed high within-group similarity and clear separation between groups, indicating distinct metabolic differences (Figure 6A). Based on the criteria of fold change (FC) ≥ 1.5 and *p* < 0.05, seven differential metabolites were identified: Sulfide, allyl methyl;4-Heptanone; Disulfide, dimethyl; Decane, 2,2-dimethyl-; Nonane; Cyclohexanone; and Hexanal (Figure 6B). Among these, cyclohexanone was again identified as a differential metabolite, with its relative abundance significantly higher in the periodontitis group (Figure 6C).

### 3.4 Detection of metabolites in saliva by LC-MS

#### 3.4.1 Analysis of saliva samples

**Figure 7:**
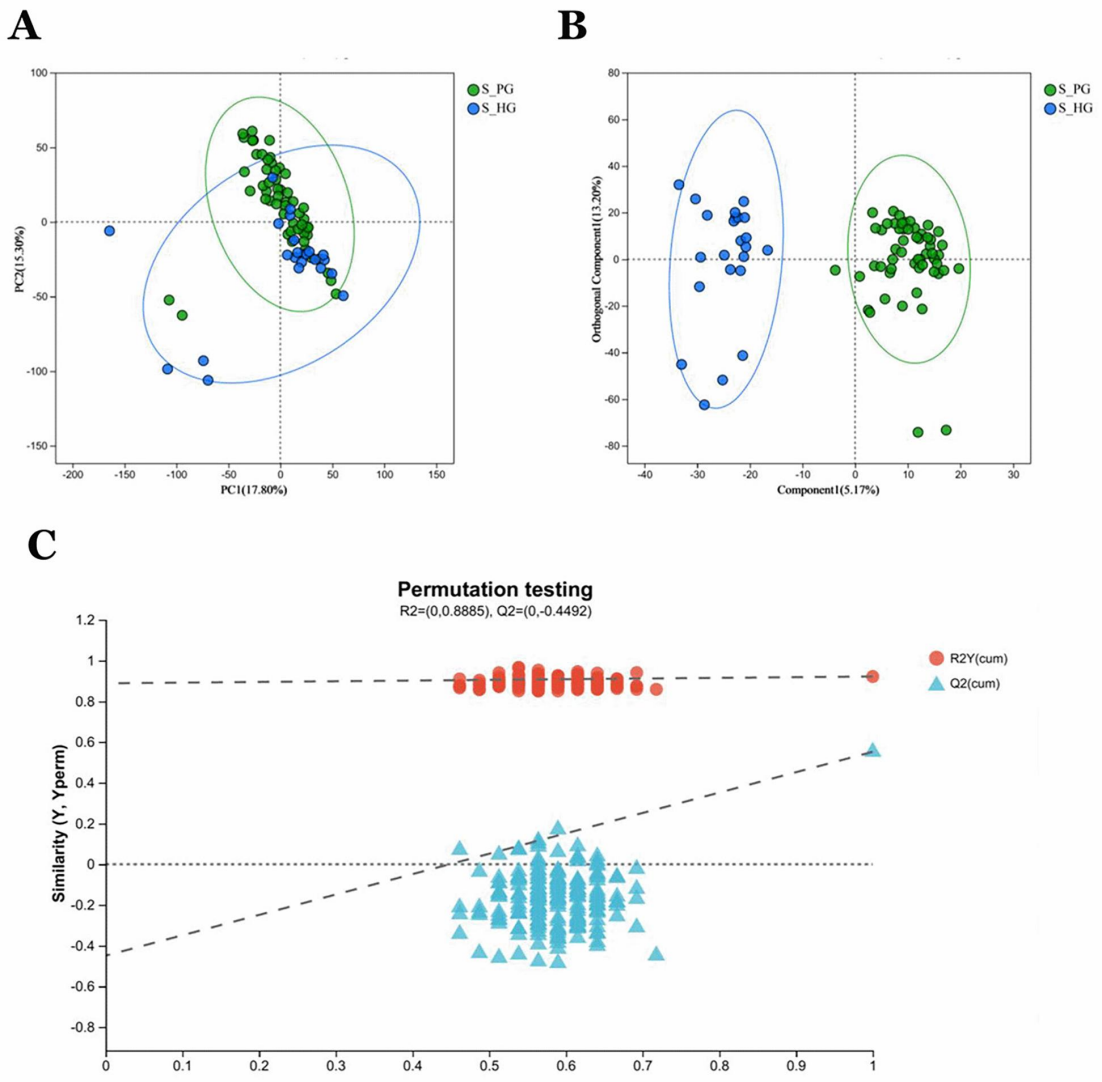
Saliva validation cohort 2: Saliva samples from periodontitis group (*n* = 55) and healthy group (*n* = 23) **(A)** PCA analysis of sample relationships. Green dots indicate S_P group, blue dots indicate S_H group. **(B)** OPLS-DA analysis of sample relationship. **(C)** Permutation testing of OPLS-DA: R2 = (0, 0.8885), Q2 = (0, −0.4492).

PCA analysis revealed that the two groups of samples, S_P and S_H, exhibited relatively small distances within each group, indicating good reproducibility of the samples (Figure 7A). Significant differences between the groups were observed in OPLS-DA analysis (Figure 7B). The degree of model explanation (R2Y) and prediction (Q2) were 0.922 and 0.552, respectively, indicating strong model performance. To ensure the reliability of the results, the OPLS-DA model was validated by a 200-permutation test. The intercept of the Q2 regression line was −0.4492, which is less than 0, confirming that the model was not overfitted (Figure 7C).

#### 3.4.2 Differential metabolite screening and pathway analysis

**Figure 8:**
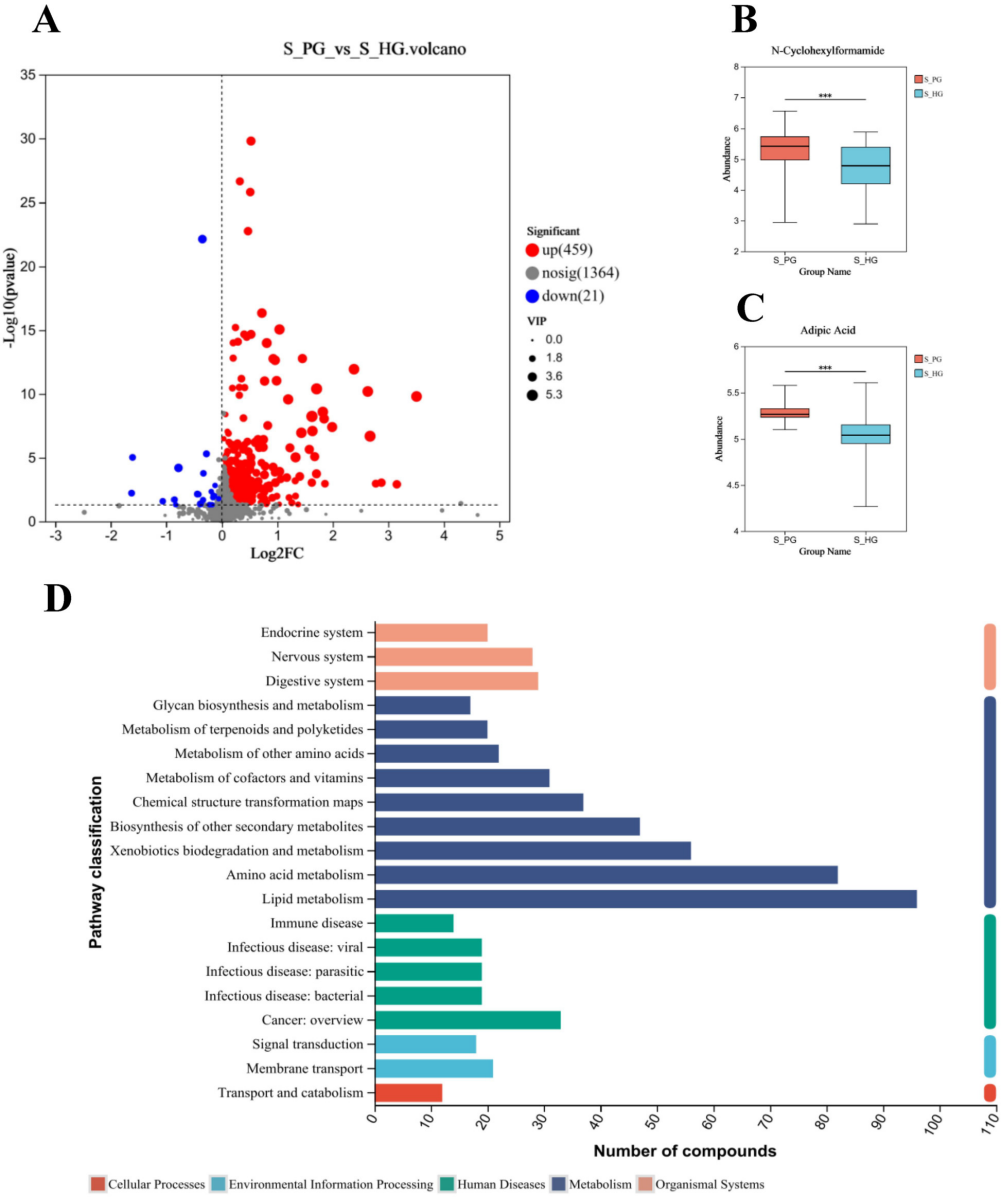
Screening and pathway analysis of differential metabolites in Saliva. **(A)** Volcano diagram: blue spot indicates the downregulated differently expressed protein, while red spot indicates the upregulated. Gray spot represents the proteins with no different expression. Screening conditions for VIP ≥ 1 and *P*-value ≤ 0.05 for OPLS-DA models. **(B)** Histogram of N-Cyclohexylformamide. **(C)** Histogram of Adipic Acid. **(D)** The vertical coordinate is the secondary classification of KEGG metabolic pathways, and the horizontal coordinate is the number of metabolites annotated to the pathway (Metabolism), Genetic Information Processing), (Environmental Information Processing), (Cellular Processes), (Organismal Systems), (Human Diseases), (Drug Development).

**Figure 9:**
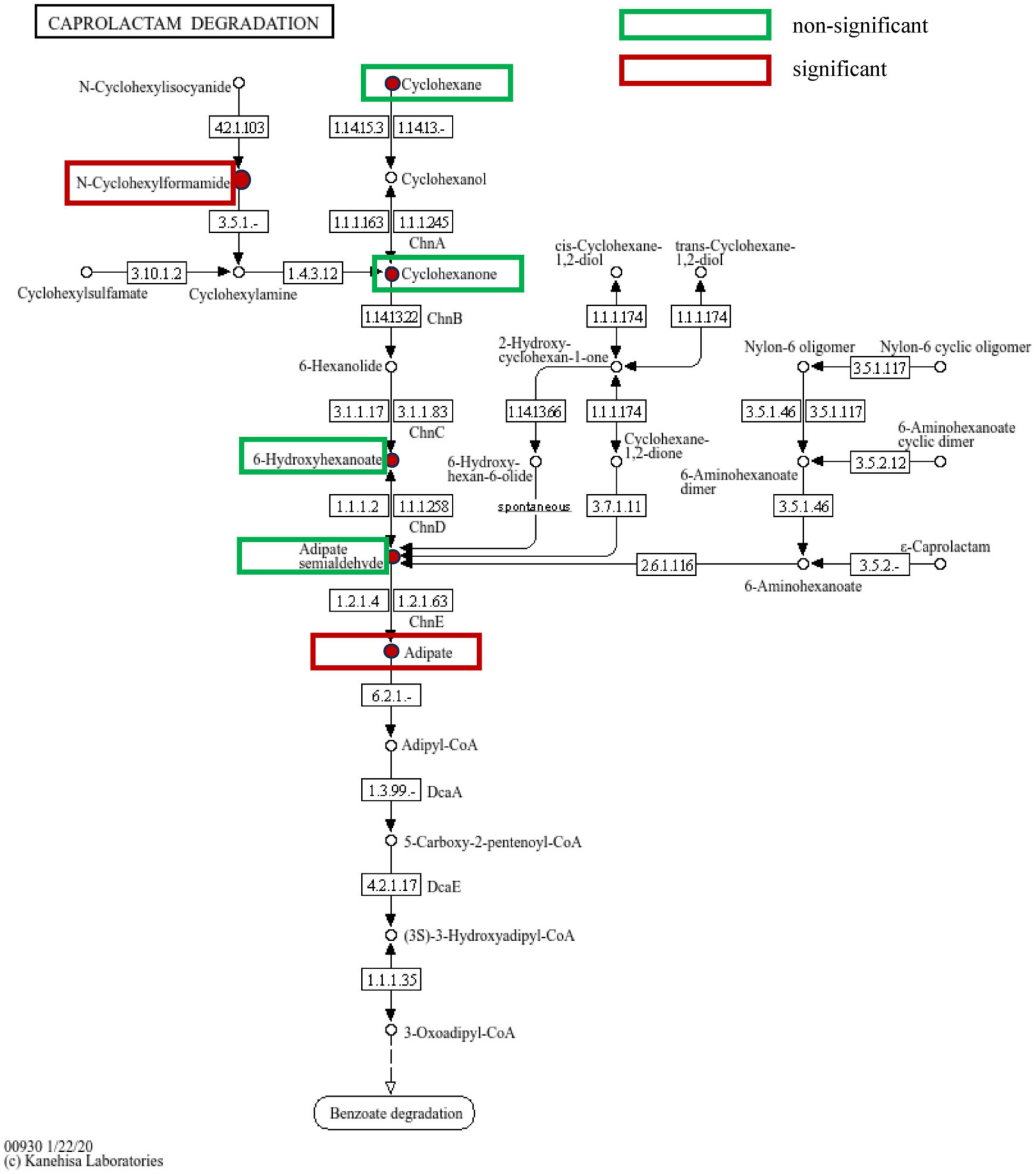
Caprolactam degradation pathway: green boxes indicate metabolites detected on the pathway but with no statistically significant differences between groups, while red boxes indicate metabolites detected and highly expressed on the pathway with statistically significant differences between groups.

A total of 1,844 metabolites were identified in saliva validation cohort 2. Differential metabolites were selected based on VIP scores from the OPLS-DA model and *p*-values from Student's t-test, with metabolites meeting the criteria of VIP > 1 and *p* < 0.05 considered statistically significant. This resulted in the identification of 480 differential metabolites (Figure 8A).Totally, 262 metabolites were mapped to various KEGG pathways, primarily related to lipid metabolism, amino acid metabolism, and microbial metabolism in different environments (Figure 8D). While styrene and cyclohexanone were detected in saliva, their differences between the periodontitis and healthy groups were not statistically significant (*p* > 0.05), and ethanol was not detected in saliva. Notably, cyclohexanone was mapped to the “map0930: caprolactam degradation pathway,” where six metabolites were detected: cyclohexane, cyclohexanone, 6-hydroxyhexanoic acid, N-cyclohexylformamide, adipic acid, and adipate semialdehyde. Among these, N-cyclohexylformamide (Figure 8B) and adipic acid (Figure 8C) exhibited statistically significant differences between groups, with higher relative abundance in the saliva of periodontitis patients. As upstream and downstream metabolites of cyclohexanone, their elevated levels suggest that the caprolactam degradation pathway might play a role in the metabolic alterations observed in periodontitis patients (Figure 9).

## 4 Discussion

In this study, we analyzed the exhalation profiles of individuals with periodontitis and healthy controls from a discovery cohort by TD-GC-MS. After screening and validating a new cohort through machine learning with a “randomized forests” approach, we developed a diagnostic model consisting of cyclohexanone, styrene, and ethanol. These three metabolites were found to be present in the volatile metabolites of saliva, confirmed by SPME-GC-MS. Cyclohexanone exhibited a consistent trend in both saliva and exhaled breath, with statistically significant differences between the groups. Additionally, the analysis of salivary metabolites revealed a link between cyclohexanone and the caprolactam degradation pathway.

Previous studies have established volatile sulfur compounds (VSCs) as key contributors to oral malodor, often linked to Gram-negative anaerobic bacteria associated with periodontitis. VSCs have been proposed as potential biomarkers for periodontitis-related oral malodor in adults (26). In our study, we also observed a statistically significant increase in dimethyl sulfide concentration within the periodontitis group through SPME analysis volatile metabolites in saliva. However, no significant differences in sulfides were detected in the gas samples, possibly due to the concentration of sulfides being too low in the gas samples or the presence of oxidation prior to sample processing. This finding is consistent with that of another research group, which observed a lower detection rate of volatile metabolites in exhaled breath compared to urine and other liquid samples (27). Previous research has suggested that periodontal disease is associated with an increased risk of halitosis. However, not all periodontitis patients exhibit oral malodor, and VSCs are not directly applicable as biomarkers for periodontitis itself (28). Therefore, further investigation into the relationship between periodontitis and sulfides is warranted by improving the sampling methods and expanding the sample size.

In this study, ethanol is one of the volatile metabolites in the diagnostic model. Ethanol is a volatile organic compound in saliva, produced by bacterial alcohol dehydrogenase or alcohol consumption, and can be converted to acetaldehyde (29). In a previous study using nuclear magnetic resonance spectroscopy, ethanol was identified as a biomarker for periodontitis. Interestingly, the ethanol concentration was lower in periodontitis patients compared to healthy controls, suggesting a link to microbial oxidative activity in periodontitis (5). This discrepancy may be attributed to differences in detection methods and the relatively low ethanol concentration in saliva. It is possible that ethanol is more likely to exist in the form of volatile metabolites in exhaled breath rather than in saliva.

Styrene, a volatile organic compound (VOC) that has been linked to increased lung cancer risk, has been extensively studied and identified as a promising biomarker for various health conditions (30). Recently, a research team employed needle trap extraction to analyze volatile compounds present above Helicobacter pylori cultures. They revealed that styrene was markedly elevated in the culture medium of Helicobacter pylori. This phenomenon, along with other notably distinct metabolites, contributed to a unique volatile profile that could as a potential tool for monitoring infections caused by this pathogen (31). In our study, styrene levels were significantly elevated in the periodontitis group, suggesting a potential link to oral microbiome dysbiosis.

Cyclohexanone (CAS number: 108-94-1), a six-carbon cyclic compound with a ketone group, was identified as a potential volatile biomarker for periodontitis in this study. Cyclohexanone has been detected in previous research as a VOC associated with various diseases. It has been found in the breath of patients with colorectal cancer (32), and elevated levels have been observed in patients with chronic kidney disease following hemodialysis (33). A research team had identified cyclohexanone as a promising biomarker in the breath of patients with biliary diseases, as well as in the volatile gases present in bile (34, 35). Our study excluded systemic diseases using strict inclusion criteria, suggesting that the elevated levels of cyclohexanone in periodontitis patients are primarily due to an imbalance in the oral microbiome. Another research team measured the components of VOCs by using Proton Transfer Reaction Time-of-Flight Mass Spectrometry (MS). They found the cyclohexanone in oral volatile organic compounds will decrease by an average of 4.3% among healthy individuals after brushing their teeth every morning (36). It indicates that the detection rate of cyclohexanone will increase in the more complex oral microbiota environment, further demonstrating that patients with periodontitis have a dysbiosis of the oral microbiota, metabolic disorders, leading to an increase in cyclohexanone.

Our study revealed a significant increase in cyclohexanone concentration among salivary volatile metabolites in patients with periodontitis, with consistent detection through both TD-GC-MS and SPME-GC-MS analyses. Furthermore, the cyclohexanone was annotated to the caprolactam metabolic pathway along with the other five differential metabolite analysis of saliva. Therefore, we hypothesized that cyclohexanone in oral exhaled breath mainly originated from the caprolactam degradation pathway in saliva of periodontitis patients. The caprolactam degradation pathway has been regarded as an ex vivo metabolic pathway for different microbial metabolisms. However, in recent years, it has been found to play an important role in systemic diseases. We speculate that this metabolic pathway may also be an endogenous metabolic pathway. A research team analyzed the progression of nasal microbiota detection in young adults and elderly asthmatics using high-throughput sequencing, and found that the relative abundance of lysine degradation, N-glycan biosynthesis, caprolactam degradation, and PPAR signaling pathways was significantly lower in asthmatics than in non-asthmatics, which may be associated with the reduction of inflammation and degradation of air pollutants (37). In our research, N-Cyclohexylformamide had a rise in expression in the periodontitis group. It is at the upstream of the caprolactam degradation pathway. With the increase of metabolites at the upstream, the cyclohexanone in the downstream will also increase. Due to cyclohexanone slightly water-soluble and easily volatilizable properties, it is more often detected in the gaseous form.

The team of Kajsa Roslund employed a combination of solid-phase microextraction (SPME) and offline gas chromatography-mass spectrometry (GC-MS) to measure volatile compounds produced by bacteria. They analyzed the *in vitro* volatile fingerprints of several major pathogens associated with periodontitis, including Porphyromonas gingivalis ATCC 33277, P. gingivalis ATCC 53978 (W50), P. gingivalis OMG434, Prevotella intermedia ATCC 25611, Prevotella nigrescens ATCC 35563, and Tannerella forsythia ATCC 43037. Signals for ethanol and styrene were detected above cultures of these periodontitis-related microbes grown on agar medium. Specifically, the ethanol signal intensified at 90 h compared to 34 h for P. gingivalis W50 and P. gingivalis OMG434. While the styrene signal showed no significant variation above the three serotypes of P. gingivalis, it increased at 90 h for P. nigrescens ATCC 35563 and T. forsythia ATCC 43037. However, the study noted that these two volatile organic compounds (VOCs) are not necessarily produced directly by the bacteria, and the precise mechanisms were not investigated in detail. Although cyclohexanone itself was not directly detected in this study, propylcyclohexane was found to be produced by P. gingivalis OMG434 (38). In a prior study by the same team utilizing Proton Transfer Reaction Time-of-Flight Mass Spectrometry (PTR-TOF-MS) to analyze these periodontitis-associated microorganisms, all three P. gingivalis strains appeared to produce high levels of acetone, suggesting its potential as a major marker for this bacterium (39). Ketone formation can be attributed to two possible pathways: the oxidation of secondary alcohols catalyzed either by alcohol dehydrogenase (ADH) or by cytochrome P450 CYP2E1 (40). Acetone can be generated via the ADH-catalyzed oxidation of 2-propanol, whereas the biosynthetic pathway for cyclohexanone involves a two-stage enzymatic reaction: initial hydroxylation of cyclohexane to cyclohexanol by bacterial cyclohexane monooxygenase, followed by further oxidation to cyclohexanone by cyclohexanol dehydrogenase. The present study identified the presence of cyclohexane in the saliva of periodontitis patients, along with significantly elevated concentrations of cyclohexanone in both exhaled breath and salivary volatile metabolites. We hypothesize that the dysbiosis induced by periodontitis may activate alkane metabolic pathways at the mechanistic level, thereby promoting the cyclohexane-cyclohexanol-cyclohexanone cascade. Notably, this metabolic axis coincides with key nodes of the caprolactam degradation pathway annotated in the KEGG database, suggesting that oral microbes might influence the host-microbe metabolic interaction network by modulating this pathway. These observations collectively provide a novel metabolic perspective for elucidating the pathological mechanisms of periodontitis and indicate that targeted intervention in the caprolactam degradation pathway could emerge as a crucial regulatory node for novel periodontitis treatment strategies.

This study has several limitations that warrant consideration. Firstly, strict screening conditions increased the difficulty of collecting healthy samples. The prevalence of subjects with periodontal health was 5% based on data from the Fourth National Oral Health Survey of China (41). It was the main reason for failing to match a sufficient number of periodontal health samples of older adults in this study. Secondly, while our metabolomic findings implicate the caprolactam degradation pathway in periodontitis, a key mechanistic limitation of our study is the inability to pinpoint the precise microbial sources of critical metabolites, such as N-cyclohexylformamide. The absence of supplementary microbial culture or metagenomic sequencing data prevents us from identifying the specific oral bacteria responsible for driving this pathway and clarifying the causal relationship between oral dysbiosis and the observed metabolic shifts. Therefore, future research integrating culturomics with metagenomic analyses is essential to isolate the relevant microorganisms and characterize the functional genes underlying this pathway's activation in periodontitis.

## 5 Conclusions

We have developed a novel method for collecting and analyzing salivary volatile metabolites by TD-GC-MS. This method holds significant promise for diagnosing periodontitis. Our findings indicate that the diagnostic model, constructed from volatile metabolites such as cyclohexanone, ethanol, and styrene in saliva, demonstrates strong potential for periodontitis diagnosis. Furthermore, the caprolactam degradation pathway associated with cyclohexanone may play a crucial role in future studies investigating oral microbiota dysbiosis in periodontitis patients.

## Data Availability

The raw data supporting the conclusions of this article will be made available by the authors, without undue reservation.

## Appendix 1: Analysis of saliva sample using LC-MS

### Quality control sample

As a part of the system conditioning and quality control process, a pooled quality control sample (QC) was prepared by mixing equal volumes of all samples. The QC samples were disposed and tested in the same manner as the analytic samples. It helped to represent the whole sample set, which would be injected at regular intervals (every 5–15 samples) in order to monitor the stability of the analysis.

### (UHPLC-MS/MS) analysis

The LC-MS/MS analysis of sample was conducted on a Thermo UHPLC-Q Exactive HF-X system equipped with an ACQUITY HSS T3 column (100 mm × 2.1 mm i.d., 1.8 μm; Waters, USA) at Majorbio Bio-Pharm Technology Co. Ltd. (Shanghai, China). The mobile phases consisted of 0.1% formic acid in water:acetonitrile (95:5, v/v) (solvent A) and 0.1% formic acid in acetonitrile:isopropanol:water (47.5:47.5, v/v) (solvent B). Positive ion mode separation gradient: 0–3 min, mobile phase B was increased from 0% to 20%; 3–4.5 min, mobile phase B was increased from 20% to 35%; 4.5–5 min, mobile phase B was increased from 35% to 100%; 5–6.3 min, mobile phase B was maintained at 100%; 6.3–6.4 min, mobile phase B was decreased from 100% to 0%; 6.4–8 min, mobile phase B was maintained at 0%. Separation gradient in negative ion mode: 0–1.5 min, mobile phase B rises from 0 to 5%; 1.5–2 min, mobile phase B rises from 5% to 10%; 2–4.5 min, mobile phase B rises from 10% to 30%; 4.5–5 min, mobile phase B rises from 30% to 100%; 5–6.3 min, mobile phase B linearly maintains 100%; 6.3–6.4 min, the mobile phase B decreased from 100% to 0%; 6.4–8 min, the mobile phase B was linearly maintained at 0%. The flow rate was 0.40 mL/min and the column temperature was 40℃.

MS conditions:

The mass spectrometric data were collected using a Thermo UHPLC-Q Exactive HF-X Mass Spectrometer equipped with an electrospray ionization (ESI) source operating in positive mode and negative mode. The optimal conditions were set as followed: source temperature at 425℃; sheath gas flow rate at 50 arb; Aux gas flow rate at 13 arb; ion-spray voltage floating (ISVF) at −3,500 V in negative mode and 3,500 V in positive mode, respectively; Normalized collision energy, 20–40–60 V rolling for MS/MS. Full MS resolution was 60,000, and MS/MS resolution was 7,500. Data acquisition was performed with the Data Dependent Acquisition (DDA) mode. The detection was carried out over a mass range of 70–1,050 m/z.

### Analysis of the correlation between differential metabolites and age

**Table A1:** Analysis of the correlation between differential metabolites and age in the periodontitis group.

Metabolites	*R*	*R* ^2^	Adjusted *R*^2^	*P*
2-Ethyl-oxetane	0.037	0.001	−0.008	0.708
Butanoic acid, methyl ester	0.063	0.004	−0.006	0.527
2-Pyrrolidinone, 1-methyl-	0.188	0.035	0.026	0.057
Ethanol, 2-butoxy-	0.033	0.001	−0.009	0.741
Ethanol	0.135	0.018	0.009	0.173
Cyclopentane, methyl-	0.031	0.001	−0.009	0.758
Decane	0.165	0.027	0.018	0.096
2,4-Dimethyl-1-heptene	0.237	0.056	0.047	0.016
Butylated Hydroxytoluene	0.090	0.008	−0.002	0.367
Undecane, 3-methyl-	0.055	0.003	−0.007	0.578
Cyclohexanone	0.071	0.005	−0.005	0.475
2,3-Butanedione	0.035	0.001	−0.009	0.725
Styrene	0.114	0.013	0.003	0.252
Propanoic acid, 2-methyl-, 1-(1,1-dimethylethyl)-2-methyl-1,3-propanediyl ester	0.164	0.027	0.017	0.097

**Table A2:** Analysis of the correlation between differential metabolites and age in the healthy group.

Metabolites	*R*	*R* ^2^	Adjusted *R*^2^	*P*
2-Ethyl-oxetane	0.733	0.537	0.515	<0.001
Butanoic acid, methyl ester	0.425	0.181	0.142	0.053
2-Pyrrolidinone, 1-methyl-	0.219	0.048	0.003	0.315
Ethanol, 2-butoxy-	0.140	0.019	−0.027	0.525
Ethanol	0.402	0.162	0.122	0.057
Cyclopentane, methyl-	0.715	0.512	0.489	<0.001
Decane	0.018	0.001	−0.470	0.935
2,4-Dimethyl-1-heptene	0.385	0.148	0.108	0.069
Butylated Hydroxytoluene	0.248	0.061	0.017	0.254
Undecane, 3-methyl-	0.305	0.093	0.050	0.157
Cyclohexanone	0.025	0.001	0.043	0.905
2,3-Butanedione	0.571	0.326	0.297	0.003
Styrene	0.145	0.021	−0.022	0.490
Propanoic acid, 2-methyl-, 1-(1,1-dimethylethyl)-2-methyl-1,3-propanediyl ester	0.013	<0.001	−0.043	0.950

*R*: Strength and direction of the linear correlation between the independent and dependent variables.

*R*-squared (*R*^2^): Proportion of variance explained by the model.

Adjusted *R*-squared: *R*-squared adjusted for degrees of freedom, to prevent overfitting.

*P*-value: Tests whether the independent variable has a significant effect on the dependent variable.

## References

[1] Kinane DF Stathopoulou PG Papapanou PN. Periodontal diseases. Nat Rev Dis Primer. (2017) 3:17038. 10.1038/nrdp.2017.38 28805207

[2] Peres MA Macpherson LMD Weyant RJ Daly B Venturelli R Mathur MR Oral diseases: a global public health challenge. Lancet. (2019) 394(10194):249–60. 10.1016/S0140-6736(19)31146-8 31327369

[3] Bernabe E Marcenes W Hernandez CR Bailey J Abreu LG Alipour V Global, regional, and national levels and trends in burden of oral conditions from 1990 to 2017: a systematic analysis for the global burden of disease 2017 study. J Dent Res. (2020) 99(4):362–73. 10.1177/0022034520908533 32122215 PMC7088322

[4] Kaczor-Urbanowicz KE Carreras-Presas CM Aro K Tu M Garcia-Godoy F Wong DT. Saliva diagnostics – current views and directions. Exp Biol Med. (2017) 242(5):459. 10.1177/1535370216681550 PMC5367650 27903834

[5] Kim S Kim H-J Song Y Lee HA Kim S Chung J. Metabolic phenotyping of saliva to identify possible biomarkers of periodontitis using proton nuclear magnetic resonance. J Clin Periodontol. (2021) 48(9):1240–9. 10.1111/jcpe.13516 34189748

[6] Lasisi TJ Lawal FB. Preference of saliva over other body fluids as samples for clinical and laboratory investigations among healthcare workers in Ibadan, Nigeria. Pan Afr Med J. (2019) 34:191. 10.11604/pamj.2019.34.191.18738 32180865 PMC7060920

[7] Gardner A Carpenter G So P-W. Salivary metabolomics: from diagnostic biomarker discovery to investigating biological function. Metabolites. (2020) 10(2):47. 10.3390/metabo10020047 31991929 PMC7073850

[8] Nazar NSBM Ramanathan A Ghani WMN Rokhani FB Jacob PS Sabri NEB Salivary metabolomics in oral potentially malignant disorders and oral cancer patients-a systematic review with meta-analysis. Clin Oral Investig. (2024) 28(1):98. 10.1007/s00784-023-05481-6 38225483

[9] Nijakowski K Owecki W Jankowski J Surdacka A. Salivary biomarkers for Alzheimer’s disease: a systematic review with meta-analysis. Int J Mol Sci. (2024) 25(2):1168. 10.3390/ijms25021168 38256241 PMC10817083

[10] Sahibzada HA Khurshid Z Khan RS Naseem M Siddique KM Mali M Salivary IL-8, IL-6 and TNF-α as potential diagnostic biomarkers for oral cancer. Diagn Basel Switz. (2017) 7(2):21. 10.3390/diagnostics7020021 PMC5489941 28397778

[11] Liebsch C Pitchika V Pink C Samietz S Kastenmüller G Artati A The Saliva metabolome in association to oral health Status. J Dent Res. (2019) 98(6):642–51. 10.1177/0022034519842853 31026179

[12] Phillips M Bauer TL Pass HI. A volatile biomarker in breath predicts lung cancer and pulmonary nodules. J Breath Res. (2019) 13(3):036013. 10.1088/1752-7163/ab21aa 31085817

[13] van der Schee MP Paff T Brinkman P van Aalderen WMC Haarman EG Sterk PJ. Breathomics in lung disease. Chest. (2015) 147(1):224–31. 10.1378/chest.14-0781 25560860

[14] Jia Z Zhang H Ong CN Patra A Lu Y Lim CT Detection of lung cancer: concomitant volatile organic compounds and metabolomic profiling of six cancer cell lines of different histological origins. ACS Omega. (2018) 3(5):5131–40. 10.1021/acsomega.7b02035 30023907 PMC6044508

[15] Tiele A Wicaksono A Kansara J Arasaradnam RP Covington JA. Breath analysis using eNose and Ion mobility technology to diagnose inflammatory bowel disease-A pilot study. Biosensors. (2019) 9(2):55. 10.3390/bios9020055 31013848 PMC6627846

[16] Tong H Wang Y Li Y Liu S Chi C Liu D Volatile organic metabolites identify patients with gastric carcinoma, gastric ulcer, or gastritis and control patients. Cancer Cell Int. (2017) 17:108. 10.1186/s12935-017-0475-x 29200968 PMC5699190

[17] Minh TDC Blake DR Galassetti PR. The clinical potential of exhaled breath analysis for diabetes mellitus. Diabetes Res Clin Pract. (2012) 97(2):195–205. 10.1016/j.diabres.2012.02.006 22410396 PMC3384765

[18] Haiek M Dvoyris V Broza YY Haick H Weiss E Houri-Haddad Y. Bacterial volatile organic compounds as potential caries and periodontitis disease biomarkers. Int J Mol Sci. (2025) 26(8):3591. 10.3390/ijms26083591 40332108 PMC12027193

[19] Magrin GL Strauss FJ Benfatti CAM Maia LC Gruber R. Effects of short-chain fatty acids on human oral epithelial cells and the potential impact on periodontal disease: a systematic review of *in vitro* studies. Int J Mol Sci. (2020) 21(14):4895. 10.3390/ijms21144895 32664466 PMC7402343

[20] Yu X Shahir AM Sha J Feng Z Eapen B Nithianantham S Short-Chain fatty acids from periodontal pathogens suppress histone deacetylases, EZH2, and SUV39H1 to promote Kaposi’s sarcoma-associated herpesvirus replication. J Virol. (2014) 88(8):4466–79. 10.1128/JVI.03326-13 24501407 PMC3993761

[21] Milanowski M Pomastowski P Ligor T Buszewski B. Saliva - volatile biomarkers and profiles. Crit Rev Anal Chem. (2017) 47(3):251–66. 10.1080/10408347.2016.1266925 27905825

[22] Ge D Zhou J Chu Y Lu Y Zou X Xia L Distinguish oral-source VOCs and control their potential impact on breath biomarkers. Anal Bioanal Chem. (2022) 414(6):2275–84. 10.1007/s00216-021-03866-8 34982180

[23] Ulrich S. Solid-phase microextraction in biomedical analysis. J Chromatogr A. (2000) 902(1):167–94. 10.1016/S0021-9673(00)00934-1 11192153

[24] Diao J Yuan C Tong P Ma Z Sun X Zheng S. Potential roles of the free salivary microbiome dysbiosis in periodontal diseases. Front Cell Infect Microbiol. (2021) 11:711282. 10.3389/fcimb.2021.711282 34631597 PMC8493099

[25] Caton JG Armitage G Berglundh T Chapple ILC Jepsen S Kornman KS A new classification scheme for periodontal and peri-implant diseases and conditions - Introduction and key changes from the 1999 classification. J Clin Periodontol. (2018) 45(Suppl 20):S1–8. 10.1111/jcpe.12935 29926489

[26] Morita M Wang H-L. Association between oral malodor and adult periodontitis: a review. J Clin Periodontol. (2001) 28(9):813–9. 10.1034/j.1600-051x.2001.028009813.x 11493349

[27] Amann A Costello Bde L Miekisch W Schubert J Buszewski B Pleil J The human volatilome: volatile organic compounds (VOCs) in exhaled breath, skin emanations, urine, feces and saliva. J Breath Res. (2014) 8(3):034001. 10.1088/1752-7155/8/3/034001 24946087

[28] Lee Y-H Shin S-I Hong J-Y. Investigation of volatile sulfur compound level and halitosis in patients with gingivitis and periodontitis. Sci Rep. (2023) 13:13175. 10.1038/s41598-023-40391-3 37580412 PMC10425441

[29] Homann N Tillonen J Rintamäki H Salaspuro M Lindqvist C Meurman JH. Poor dental status increases acetaldehyde production from ethanol in saliva: a possible link to increased oral cancer risk among heavy drinkers. Oral Oncol. (2001) 37(2):153–8. 10.1016/s1368-8375(00)00076-2 11167142

[30] Saalberg Y Wolff M. VOC Breath biomarkers in lung cancer. Clin Chim Acta Int J Clin Chem. (2016) 459:5–9. 10.1016/j.cca.2016.05.013 27221203

[31] Vangravs R Mežmale L Slefarska-Wolak D Dauss E Ager C Corvalan AH Volatilomic signatures of different strains of Helicobacter pylori. Helicobacter. (2024) 29(2):e13064. 10.1111/hel.13064 38459689

[32] Kononova E Mežmale L Polaka I Veliks V Anarkulova L Vilkoite I Breath fingerprint of colorectal cancer patients based on the gas chromatography–mass spectrometry analysis. Int J Mol Sci. (2024) 25(3):1632. 10.3390/ijms25031632 38338911 PMC10855950

[33] Seong S-H Kim HS Lee Y-M Kim J-S Park S Oh J. Exploration of potential breath biomarkers of chronic kidney disease through thermal desorption–gas chromatography/mass spectrometry. Metabolites. (2023) 13(7):837. 10.3390/metabo13070837 37512544 PMC10385797

[34] Zhang X Gui X Zhang Y Liu Q Zhao L Gao J A panel of bile volatile organic compounds servers as a potential diagnostic biomarker for gallbladder cancer. Front Oncol. (2022) 12:858639. 10.3389/fonc.2022.858639 35433420 PMC9006947

[35] Gui X Zhang X Xin Y Liu Q Wang Y Zhang Y Identification and validation of volatile organic compounds in bile for differential diagnosis of perihilar cholangiocarcinoma. Clin Chim Acta Int J Clin Chem. (2023) 541:117235. 10.1016/j.cca.2023.117235 36716909

[36] Ratiu IA Ligor T Bocos-Bintintan V Mayhew CA Buszewski B. Volatile organic compounds in exhaled breath as fingerprints of lung cancer, asthma and COPD. J Clin Med. (2020) 10(1):32. 10.3390/jcm10010032 33374433 PMC7796324

[37] Lee JJ Kim SH Lee MJ Kim BK Song WJ Park HW Different upper airway microbiome and their functional genes associated with asthma in young adults and elderly individuals. Allergy. (2019) 74(4):709–19. 10.1111/all.13608 30242844

[38] Roslund K Lehto M Pussinen P Hartonen K Groop PH Halonen L Identifying volatile *in vitro* biomarkers for oral bacteria with proton-transfer-reaction mass spectrometry and gas chromatography-mass spectrometry. Sci Rep. (2021) 11(1):16897. 10.1038/s41598-021-96287-7 34413397 PMC8377122

[39] Roslund K Lehto M Pussinen P Groop P-H Halonen L Metsälä M. On-line profiling of volatile compounds produced *in vitro* by pathogenic oral bacteria. J Breath Res. (2019) 14(1):016010. 10.1088/1752-7163/ab5559 31698353

[40] Kalapos MP. On the mammalian acetone metabolism: from chemistry to clinical implications. Biochim Biophys Acta. (2003) 1621(2):122–39. 10.1016/s0304-4165(03)00051-5 12726989

[41] Sun H Du M Tai B Chang S Wang Y Jiang H. Prevalence and associated factors of periodontal conditions among 55- to 74-year-old adults in China: results from the 4th national oral health survey. Clin Oral Investig. (2020) 24(12):4403–12. 10.1007/s00784-020-03306-4 32382923

